# Intracellular delivery of antibodies by chimeric Sesbania mosaic virus (SeMV) virus like particles

**DOI:** 10.1038/srep21803

**Published:** 2016-02-24

**Authors:** Ambily Abraham, Usha Natraj, Anjali A. Karande, Ashutosh Gulati, Mathur R. N. Murthy, Sathyabalan Murugesan, Pavithra Mukunda, Handanahal S. Savithri

**Affiliations:** 1 Department of Biochemistry, Indian Institute of Science, Karnataka, India; 2 Molecular Biophysics Unit, Indian Institute of Science, Karnataka, India; 3 Theramyt Novobiologics Pvt. Ltd., Karnataka, India

## Abstract

The therapeutic potential of antibodies has not been fully exploited as they fail to cross cell membrane. In this article, we have tested the possibility of using plant virus based nanoparticles for intracellular delivery of antibodies. For this purpose, *Sesbania mosaic virus* coat protein (CP) was genetically engineered with the B domain of *Staphylococcus aureus* protein A (SpA) at the βH-βI loop, to generate SeMV loop B (SLB), which self-assembled to virus like particles (VLPs) with 43 times higher affinity towards antibodies. CP and SLB could internalize into various types of mammalian cells and SLB could efficiently deliver three different monoclonal antibodies–D6F10 (targeting abrin), anti-α-tubulin (targeting intracellular tubulin) and Herclon (against HER2 receptor) inside the cells. Such a mode of delivery was much more effective than antibodies alone treatment. These results highlight the potential of SLB as a universal nanocarrier for intracellular delivery of antibodies.

Antibody based therapy is a successful protein targeting strategy in medicine that can disrupt protein-protein interactions or inhibit signalling pathways^1^,^2^. However, most of these antibodies are incapable of internalizing in target cells. Hence, majority of the FDA approved antibodies are those targeting surface exposed receptors. For example, Trastuzumab (Herceptin/Herclon) targeting overexpressed and surface exposed HER2 receptor is effective in the treatment of HER2 positive breast cancer patients^3^,^4^.

Internalization of antibodies has been shown to enhance the cytotoxicity of antibodies as well as minimize side effects^5^. For example, immunoliposomes targeted to CD19 have higher therapeutic efficiency as compared to those targeting surface exposed CD20^6^. There have been various attempts to internalize antibodies by fusion with protein transduction domains/cell penetrating peptides^7^ or conjugation to liposomes, polymerosomes or synthetic nanoparticles like poly L arginine, gold nanoparticles etc^8^,^9^. However, very few virus-based nanoparticles (VNPs) or virus like particles (VLPs) have been explored for such applications. Apart from animal viral vectors that express antibodies intracellularly by transduction, there are very few universal antibody delivering agents^10^. Some of the VNPs have been genetically engineered or chemically modified with *Staphylococcus aureus* protein A (SpA) or their sub-domains like B, Z or Z33, that can bind to IgGs^11^, to create chimeric VNPs^12^,^13^. In most instances, such chimeras have been used for increased sensitivity of bioassays, cellular targeting and increased immunogenicity. Eg: Lentiviral vectors with modified Sindbis envelope (carrying ZZ domain) were targeted to metastatic melanoma cells in mice^14^. Due to the immunogenicity of animal viral vectors in humans, focus is being shifted to plant VNPs/VLPs as they are known to be non-pathogenic. Recently, *Potato virus X* (PVX) VNPs chemically conjugated with Herceptin was shown to enhance antibody cytotoxicity^15^. However, the fate of the antibody in such a mode of application was not explored. Interestingly, no plant VLPs has been developed as a universal nanocarrier for antibody delivery.

Towards this, we have chosen icosahedral *Sesbania mosaic virus* (SeMV) coat protein (CP) that self assembles to form VLPs *in vitro*. Analysis of X- ray crystal structure of native SeMV^16^ and recombinant capsids^17^ (Fig. 1A) revealed that the βH-βI loop (residues 238–245) (Fig. 1B) is surface exposed both at the pentameric and hexameric interfaces in the T = 3 icosahedral particle (~30 nm). CP was genetically engineered with B domain (58 amino acids) at the midpoint (Ser 242) of βH-βI loop to form SeMV Loop B (SLB) (Fig. 1C), which also assembles into VLPs. For the first time, we show that CP VLPs as well as the chimeric VLPs can enter various mammalian cells including HeLa, BT-474, KB, B16-F10 and HMECs. Since the B domain can bind to any antibody, it was of interest to explore the possibility of chimeric VLPs delivering antibodies intracellularly. For this, three diverse monoclonal antibodies were chosen namely D6F10, anti-α-tubulin DM1A and Herclon. D6F10^18^,^19^ is a well characterized monoclonal antibody that neutralizes the toxic effects of abrin^20^,^21^,^22^, a type II ribosome inactivating protein that inhibits protein synthesis and causes apoptosis. Anti-tubulin monoclonal antibodies (DM1A) when delivered via nanoparticles sequester intracellular tubulin and disrupt the network^23^. Schematic representation of intracellular delivery of antibodies is represented in Fig. 1D. Interestingly, SLB was able to efficiently deliver all the three antibodies inside mammalian cells and most importantly, the antibodies retained their functionality inside cells. These results demonstrate that SLB can be a universal antibody delivering agent that can enhance the efficacy of therapeutic antibodies targeted to surface antigens and also pave way for delivering other antibodies that target potential intracellular targets.

## Results

### SLB self assembles into VLPs with functional B domains

CP was genetically engineered with B domain (58 amino acids) at the midpoint (Ser 242) of βH-βI loop to form SLB. When expressed in *E. coli*, CP and SLB self-assembled into VLPs as shown by sucrose gradient profile (Fig. 2A). Transmission Electron Microscopic (TEM) images (Fig. 2B) revealed an average diameter of ~37 nm for SLB VLPs unlike CP VLPs, which showed an average diameter of ~30 nm. For ease of representation, CP VLPs and SLB VLPs will be henceforth referred to as CP and SLB respectively. In order to examine whether the B domain in SLB retains its ability to bind antibodies after VLP assembly, initially western blot analysis of CP and SLB were carried out. As shown in Fig. 2C, both VLPs were able to bind to anti-CP polyclonal antibodies while only SLB was able to bind to anti-diaminopropionate ammonia lyase (DAPAL; an *E. coli* PLP-dependent enzyme) antibodies, indicating the presence of functional B domain in SLB. This was further confirmed by DAC ELISA using anti-DAPAL antibodies. In Fig. 2D, it can be seen that SLB and SpA exhibit high affinity towards anti-DAPAL antibodies while CP shows no such binding. Interestingly, SLB showed 43 times higher affinity (~80–90 antibodies/VLP) as compared to SpA, indicating that multiple functional B domains were accessible on the chimeric VLPs.

### CP and SLB can enter mammalian cells

In order to examine entry of VLPs into mammalian cells, the VLPs were initially labelled with Alexa Fluor 488 (Supplementary Fig. S1 A). CP (0.33 mg/ml) and SLB (0.98 mg/ml) were found to be conjugated with 30.5 μM and 72.3 μM Alexa 488 respectively, demonstrating efficient labelling (~>85%) of exposed lysines (three per subunit). Further, the overall structural and functional integrity of labelled VLPs were unaltered as confirmed by TEM and western blot analysis (Supplementary Fig. S1 B and C). Interestingly, when CP 488 or SLB 488 (1.58 nM) was incubated with HeLa cells for varying time intervals (Fig. 3A), both VLPs were able to enter into the cytoplasm of HeLa cells with fluorescence reaching maximum in 4–8 hours. CP 488 incubated with BSA or sheep serum could also internalize in HeLa cells (Fig. 3B) indicating that the entry of VLPs was unaffected by the presence of non-specific proteins. Competitive inhibition with unlabelled CP (10 nM) confirmed the specificity of VLP entry (Supplementary Fig. S2 A and B).

Since CP and SLB could enter HeLa cells, it was of interest to examine if these VLPs can also enter other mammalian cells. As shown in Fig. 3C, SLB could enter KB, B16-F10 (*Mus musculus* melanoma), BT-474 (mammary duct cancer cells), CB 704 (cancerous epithelial breast cells from patient) and HMECs 704 (normal human mammary epithelial cells) also, demonstrating the versatility of cellular entry by these VLPs.

### Antibody delivery

Since SLB could efficiently bind IgGs, it was of interest to examine if SLB could serve as a nanocarrier to deliver bound antibodies inside mammalian cells. For this purpose, three different monoclonal antibodies - D6F10 (anti-abrin), anti-α-tubulin and Herclon (anti-HER2 receptor) were used as cargo.

#### SLB mediated D6F10 delivery

One of the limitations of neutralizing antibodies against toxins like abrin, is that they cannot internalize by themselves and hence necessitate the use of a vehicle that can deliver antibodies inside cells. In order to test if SLB, which can enter HeLa cells (Fig. 3A), can also deliver D6F10 intracellularly, D6F10 633 pre-incubated with SLB 488 for 1 hour, was incubated with HeLa cells for 4 hours. As shown in Fig. 4A, D6F10 alone did not enter cells as no red fluorescence is observed. However, when SLB 488-D6F10 633 was incubated with HeLa cells, D6F10 was successfully internalized (Fig. 4B) as evident from the yellow fluorescence inside the cells. Similar incubation with CP failed to deliver the antibody as shown by the absence of red and yellow fluorescence due to lack of entry of D6F10 633 (Fig. 4C), confirming the ability of SLB, but not CP, to deliver the antibodies. Kinetics of D6F10 entry when bound with SLB showed maximum internalization of SLB-D6F10 between 4–8 hours (Supplementary Fig. S3 A). SLB was completely degraded by 12 hours as the green fluorescence due to SLB 488 could not be observed at this time point (Supplementary Fig. S3 A, bottom row). Confocal microscopic analysis of HeLa cells treated with SLB 488-D6F10 633 complex showed that the fluorescence intensity of D6F10 633 decreases after 8 hours (Supplementary Fig. 3 A). This was also confirmed by western blot analysis of HeLa cells incubated with SLB 488-D6F10 633 complex for 2, 4, 8, 12 and 16 hours (Supplementary Fig. 3 B). Complete degradation of antibodies was observed after 16 hours. It may be noted that SLB-D6F10 complex was formed by simple incubation of the two proteins. In order to examine whether D6F10 could be displaced in presence of other antibodies in serum, VLP-antibody complex was incubated with sheep serum when added to HeLa cells for 3 hours. Red florescence showing the entry of D6F10 633 confirmed that once bound, D6F10 was not displaced by other antibodies (Fig. 4D,E). Thus, the antibody bound to the B domain of the chimeric VLP in the SLB-antibody complex remains bound even in presence of other antibodies.

To examine the effect of the delivered antibody on the inhibition of protein synthesis caused by abrin, tritiated leucine based protein synthesis assay was performed in presence of CP, SLB, abrin, abrin-D6F10, SLB-D6F10 and CP-D6F10 (Fig. 4F). Untreated cells were taken as control (Fig. 4F bar 1). Total incorporated counts per minute for cells incubated with CP and SLB were similar to untreated cells, indicating that the entry of CP and SLB alone had no effect on protein synthesis (Fig. 4F, bars 2, 3) whereas the addition of 0.16 nM abrin (Fig. 4F, bar 4) resulted in 75% inhibition of protein synthesis. Abrin pre-incubated with D6F10 (Fig. 4F, bars 5, 6) lead to a ~2 fold rescue, which was elevated to ~2.5–3.5 fold when D6F10 was delivered by SLB nanocarriers (Fig. 4F, bar 7, 8). ANOVA followed by Tukey’s multiple comparison tests with Abrin-D6F10 (1:50) versus SLB-D6F10-Abrin (10:50:1) showed p = 0.0056, indicating that the increase in rescue of protein synthesis by D6F10 delivered via SLB is statistically significant compared to abrin pre-incubated with D6F10. As expected, no rescue from protein synthesis inhibition was observed when cells were treated with D6F10 pre-incubated with CP (Fig. 4F, bar 9), confirming that the enhanced protein synthesis is due to the antibody delivered by chimeric SLB and not CP. The increase in the rescue of protein synthesis inhibition when the antibody was delivered by SLB shows that nanocarrier mediated delivery is more effective as compared to antibody pre-bound with toxin. One of the possible reasons for such elevated rescue could be that high number of antibodies could be internalized when delivered by SLB, while in abrin-D6F10 only few D6F10 molecules that are bound to abrin (low concentration of which is used for the assay) can enter cells.

The functionality of delivered antibody was also analysed by cell cycle progression analysis of HeLa cells treated with VLP or VLP-antibody complex by PI staining method followed by FACS analysis. The percentage of dead cell population indicated that CP (Fig. 4G bar 2) and SLB (Fig. 4G bar 3) did not cause any cell death like untreated sample (Fig. 4G bar 1). As expected, abrin caused ~54% apoptosis (Fig. 4G B, bar 4) when treated for 36 hours, while abrin-D6F10 (1:50) caused 18% apoptosis (Fig. 4G, bar 5). When SLB-D6F10 complex was incubated with HeLa cells for 4 hours, followed by abrin treatment for 36 hours, the percentage of dead cells dropped to 10% (Fig. 4G B, bar 6), indicating that D6F10 delivered by SLB was functional and holds better potential in preventing abrin mediated cytotoxicity than abrin pre-incubated with D6F10. Additionally, since large number of antibodies can be internalized by SLB (due to the presence of multiple functional B domains), the efficacy of neutralizing antibody could be enhanced as shown by the augmented rescue of abrin mediated protein synthesis inhibition and apoptosis. These results validate the use of SLB as an antibody delivering agent and could widen the use of neutralizing antibodies in therapy.

#### SLB mediated anti-tubulin antibody delivery

Tubulin is an abundant cytoskeleton element and therefore delivery of anti-tubulin antibodies to specific cancer cells can effectively result in death of target cells due to disruption of tubulin network. Since SLB can deliver antibodies within cells, it was of interest to examine the effect of anti-tubulin antibody delivery by SLB in HeLa cells.

Fixed HeLa cells when immune-probed with FITC labelled anti α-tubulin antibody showed a tubular network (Fig. 5A), indicating that antibody binds to tubulin that is present in a tubular network. Antibody alone was unable to cross the membrane barrier (Fig. 5B). To test the ability of SLB to deliver FITC labelled anti-tubulin antibodies, unlabelled SLB (1.58 nM) was preincubated with antibody for 1 hour, followed by incubation with HeLa cells for 2 hours and the confocal images were captured. As evident from Fig. 5C, cytoplasmic appearance of tubulin antibodies were observed instead of a network arrangement, confirming the entry of the antibodies and disruption of the tubular network. When antibody (30 ng/μl) was delivered via SLB, aggregates similar to that reported for dioctadecylglycylspermine (DOGS) mediated anti-tubulin delivery^23^ were observed (Fig. 5D), indicating efficient disruption of tubulin network. The time course of SLB mediated anti-tubulin antibody delivery in HeLa cells (Supplementary Fig. S4) also showed an aggregation pattern after 4 hours (Supplementary Fig. S4 middle row) with an increase in size of aggregate with time as well as concentration of antibody (data not shown), indicating extensive tubulin depolymerization. These results confirm that not only were the antibodies delivered by SLB within cells, but also they were still able to interact with their specific antigen via the Fab portion of IgGs and also co-localize with the antigen. It is possible to conjugate specific cell penetrating peptides with SLB and use such chimeric VLPs to deliver anti-tubulin antibodies to cancer cells. Thus, SLB can be used for delivery of those antibodies specific to intracellular antigens that cannot cross cell membrane barriers.

#### SLB mediated Herclon delivery

In order to examine the ability of SLB to deliver therapeutic antibodies intracellularly, Herclon (Trastuzumab/Herceptin) was chosen. The antibody was initially labelled with Alexa 633 using manufacturer’s protocol and incubated with BT-474 cells (over-expressing HER2 receptor) for 1 hour. Confocal microscopic analysis of these cells indicated that Herclon localizes pre-dominantly on the surface of BT-474 cells (Fig. 6A), perhaps due to interaction with surface exposed HER2 receptors. On the other hand, addition of Herclon 633 (46 nM) pre-incubated with SLB 488 (1.58 nM) to BT-474 cells, showed red fluorescence even inside cells, revealing that SLB can deliver Herclon inside BT-474 cells (Fig. 6B). CP 488 (1.58 nM) used as control in a similar experiment failed to deliver Herclon inside BT-474 cells, although by itself CP 488 could internalize (Fig. 6C). Time course analysis also confirmed enhanced antibody delivery with increase in time of incubation as compared to antibody alone control (Supplementary Fig. S5). In order to test the specificity of Herclon, Herclon 633 (46 nM) was also incubated with HeLa cells (HER2 negative) for 2 hours and indeed there was no red fluorescence observed (Supplementary Fig. S6 A), confirming that Herclon cannot bind to cells in the absence of HER2 expression. However, as expected, SLB 488 could deliver Herclon 633 in HeLa cells as indicated by the yellow fluorescence (Supplementary Fig. S6 B).

Since SLB was able to efficiently deliver Herclon inside BT-474 cells, it was of interest to test whether the Herclon delivered by SLB could enhance the cytotoxic effects of the antibody. As expected, decreasing concentration of Herclon showed decrease in cell toxicity (Fig. 6D). Maximum cytotoxicity of 25% was observed at highest concentration of antibody (16.6 nM) when cells were treated with Herclon for 48 hours. Interestingly, SLB mediated delivery of varying concentration of Herclon showed enhanced cytotoxicity than Herclon alone (Fig. 6D). Herclon delivered via the SLB showed 83% cytotoxicity, which is 3.33 times higher than that observed with Herclon alone (p = 0.001). Further, the antibody delivered via SLB was effective even at lower concentrations (0.133 nM, 1:0.8) where there was minimal effect of Herclon alone treatment. CP and SLB by themselves had minimal effect on cell viability (Fig. 6E, bars 1 and 2) indicating that the VLPs by themselves do not cause any cytotoxic effects *in vitro*. Antibody (0.664 nM) pre-incubated with CP (Fig. 4E, bar 5) showed similar cell viability as antibody alone control (Fig. 6E, bar 3), confirming that the cytotoxic effect is due to antibodies delivered by SLB. Since SLB could also enter HeLa cells, it was of interest to examine the effect of Herclon delivered via SLB on these cells that are HER2 negative. As shown in Fig. 6E, Herclon showed no cytotoxic effect on the HeLa cells alone (Fig. 6E bar 6) or when delivered via SLB (Fig. 6E bar 7), confirming that the cytotoxic effects of Herclon is only effective in HER2 expressing cells. Thus SLB can increase the therapeutic efficiency of antibodies to surface antigens. This is the first report of a plant based chimeric VLP that enhances the toxicity of therapeutic antibody to 83% *in vitro*.

## Discussion

Bionanoparticles are being increasingly investigated as nanocarriers, as compared to synthetic nanoparticles owing to their target specificity and biocompatibility with the host. Plant viruses are particularly attractive for such applications as they are non-pathogenic in humans. Antibodies have gained importance as therapeutic molecules and are being currently administered for various diseases like cancer, autoimmune disorders etc. Intracellular antibody delivery is a major challenge in the medical field as antibodies cannot cross membrane barrier.

Plant virus nanoparticles have been extensively explored as protein cages for intracellular delivery^24^,^25^. In this paper, we have chosen the capsid protein of SeMV, a positive sense RNA virus belonging to *Sobemovirus* genus. The knowledge of the detailed structure and assembly of SeMV capsids have enabled us to identify specific positions (like the βH-βI loop) for insertion of foreign domains. Interestingly, unlike many well established plant virus nanoparticles that have to be purified via *in planta* expression^26^, SeMV capsids and chimera can be easily purified from *E.coli* with good yield. These proteins self-assemble into VLPs without the requirement of any plant host factors. SeMV CP was genetically engineered with antibody binding B domain at the midpoint of βH-βI loop to create a chimeric VLP called SeMV Loop B (SLB). Unlike chimeric *Hepatitis virus* BB capsids^11^, SLB VLPs were 7 nm wider than wild type CP VLPs. B domain, a protein with three alpha helices^27^, can bind to Fc^28^ as well as Fab regions^29^,^30^ (not close to the antigen binding site) of antibodies via its helix I and helix II. Interestingly, SLB retained its antibody binding affinity after assembly into VLPs, with 43 times more affinity as compared to SpA, suggesting that each SLB could bind as many as 80–90 antibody molecules. The K_d_ = 10 nM obtained for SLB antibody binding is similar to antibody binding observed in chimeric *Murine polyoma virus* VP1Z^31^.

For the first time, we show that CP and SLB can enter various types of mammalian cells and does not cause any toxic effects *in vitro*. *Cowpea mosaic virus* (CPMV) VNPs have been shown to enter various types of cells including endothelial cells^32^, macrophages^33^, CNS lesions^34^, atherosclerotic plaques^35^, and antigen presenting cells^36^. It enters cells by initially binding to the vimentin receptor followed by caveolar endocytosis^37^,^38^. The exact mechanism by which SeMV VLPs internalize in various cells is yet to be investigated. It has been demonstrated earlier that CPMV labelled with Fluorescein and conjugated with VEGFR1 ligand can be specifically targeted to HT-29 tumor cells in mice^39^. Thus, it is plausible that labelled CP or SLB (after conjugation with cell penetrating peptides) could also be used for such imaging techniques.

**Figure 1:**
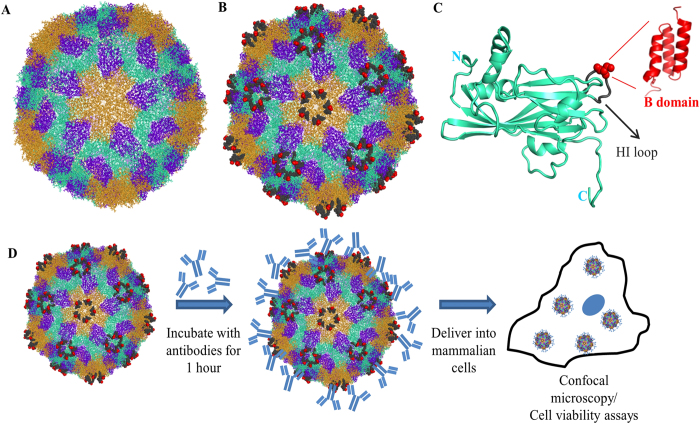
Design of SeMV CP based chimera. (**A**) Three dimensional structure of SeMV (PDB:1 × 33)^17^ showing A subunits (orange) forming pentamers and B (blue) and C (greencyan) subunits forming hexamers rendered using Pymol. The five-fold axis relating the A subunits near the centre of the particle is approximately perpendicular to the plane of illustration. (**B**) Schematic representation of SLB VLP showing the 8 residue βH-βI loops (dark grey) and Ser 242 (red spheres). (**C**) Ribbon representation of the SeMV CP C subunit showing the position of insertion of the B-domain (PDB:1SS1)^27^ at the Ser242 site (red). (**D**) Schematic representation of intracellular delivery of antibodies using SLB nanocarriers.

**Figure 2:**
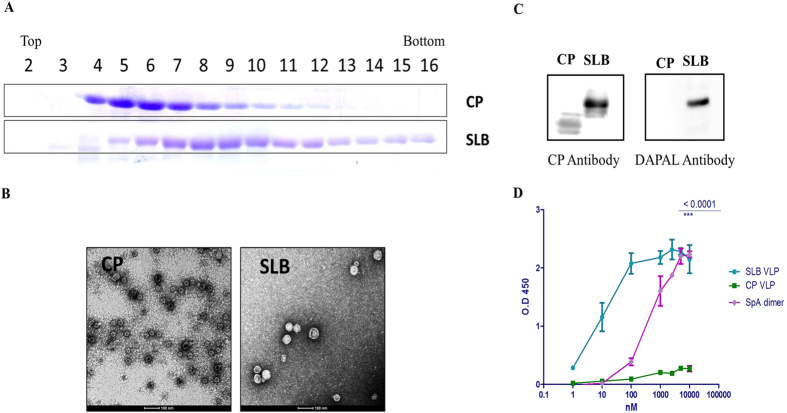
Biochemical characterization of wild type (CP) and chimeric (SLB) VLPs. (**A**) SDS PAGE analysis of 10–40% sucrose density gradient fractions (2–16) of CP (top row) and SLB (bottom row) obtained after ultracentrifugation. (**B**) Transmission electron micrographs of CP and SLB. (**C**) Western blot analysis of CP and SLB using anti-CP polyclonal antibody (left blot) and anti-DAPAL antibody (right blot). (**D**) DAC ELISA using CP VLP, SLB VLP and SpA as antigen and anti-DAPAL as primary antibody. Semilog plot of A_450_ is plotted on Y axis and varying CP, SLB and SpA (1–10000 nM) is represented on X axis.

**Figure 3:**
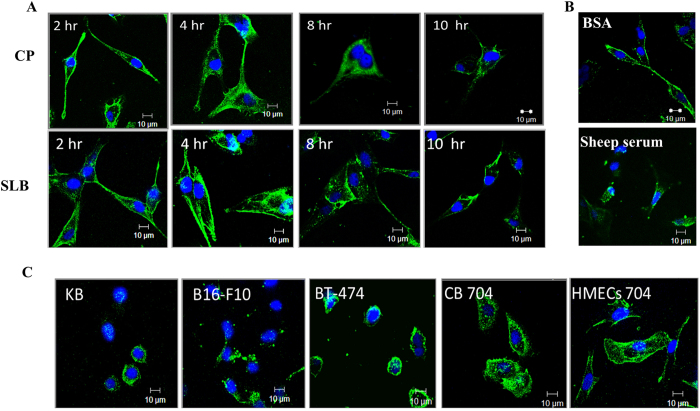
Demonstration of VLP entry in mammalian cells using confocal microscopy. (**A**) Confocal images of HeLa cells incubated with CP 488 or SLB 488 (1.58 nM) for 2, 4, 8, 10 hours at 37 °C. (**B**) Confocal images showing the entry of CP 488 (1.58 nM) in HeLa cells for 2 hours in presence of BSA/sheep serum. (**C**) Confocal images showing the entry of 1.58 nM SLB 488 in KB, B16-F10, BT-474, CB 704 and HMECs 704 cells. All confocal images were acquired using 100x/1.3 oil objective of Zeiss 510 Meta confocal microscope and analysed by LSM Image browser. Green = CP 488/SLB 488, Blue = DAPI stained nucleus

**Figure 4:**
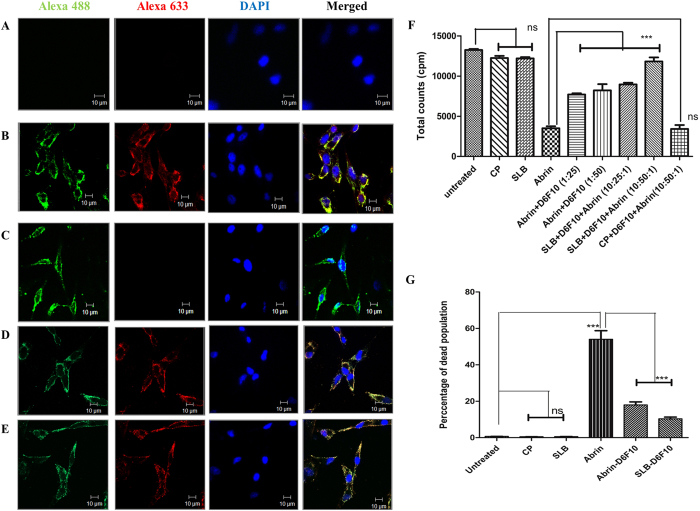
SLB mediated D6F10 delivery in HeLa cells. Confocal images of HeLa cells treated with (**A**) D6F10 633, (**B**) SLB 488-D6F10 633 and (**C**) CP 488-D6F10 633 for 4 hours. Confocal images of HeLa cells treated with SLB 488-D6F10 633 in the absence (**D**) and in the presence (**E**) of sheep serum (40 ng/μl). Green = SLB 488/CP 488, red = D6F10 633, blue = DAPI stained nuclei and Merge = all three fluorophores. (**F**) Bar diagram representing translation assay of HeLa cells treated with CP (1.5 nM), SLB (1.5 nM), abrin (0.166 nM), abrin-D6F10 (1:25, 1:50), SLB-D6F10 (10:25, 10:50) and CP. For the latter three samples, after pre-treatment of cells with SLB-D6F10 and CP-D6F10 for 2 hours, cells were treated with abrin (0.166 nM) for 7 hours. Total counts per minute (cpm) of tritium is represented on the Y axis. (**G**) Percentage of dead population obtained by cell cycle progression analysis of HeLa cells treated with CP (10 nM), SLB (10 nM), abrin (0.1 nM), abrin-D6F10 (1:50) for 36 hours and SLB-D6F10 (1:50) for 4 hours followed by abrin for 36 hours. The propidium iodide stained cells were analysed by BD FACS. ANOVA followed by Tukey’s multiple comparison tests was performed. Ns = not significant, ***p < 0.05.

**Figure 5:**
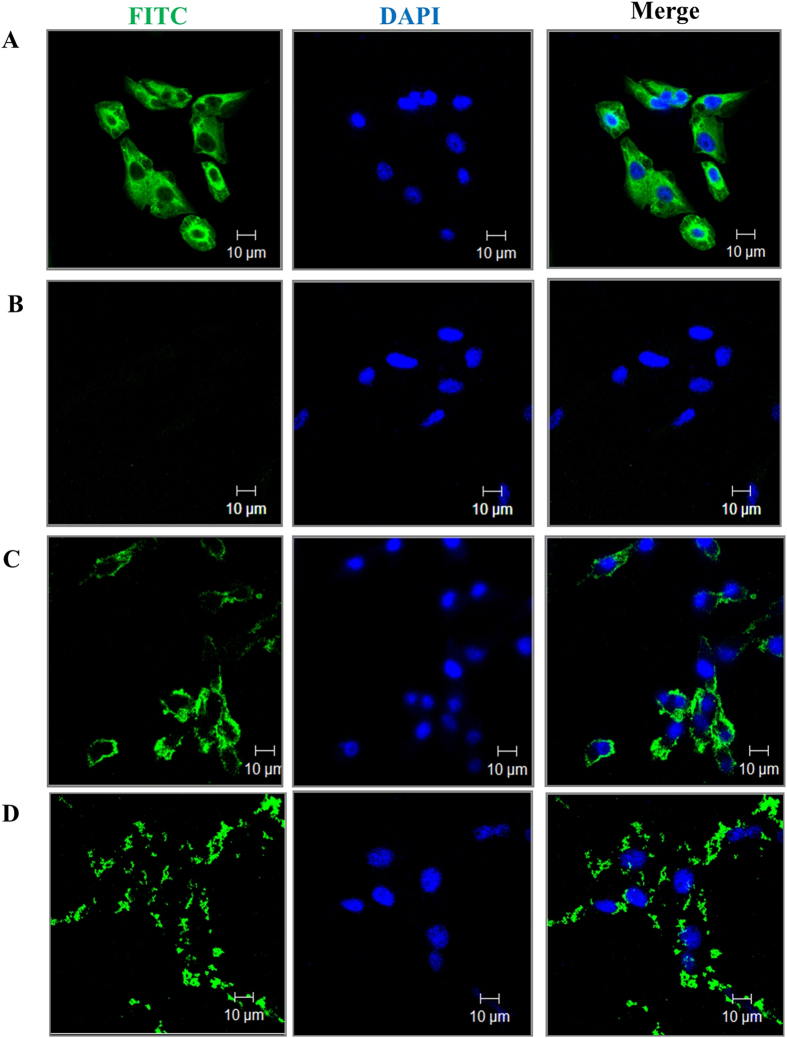
SLB mediated anti-α-tubulin antibody delivery. (**A**) Confocal images of HeLa cells immunostained with anti-α-tubulin antibody (30 ng/μl) (FITC labelled). Confocal images of HeLa cells treated with (**B**) FITC labelled anti-α-tubulin antibody (30 ng/μl), (**C**) SLB-anti-α-tubulin antibody (7.5 ng/μl) and (**D**) SLB- anti-α-tubulin antibody (30 ng/μl) for 2 hours. Green = FITC labelled tubulin antibody, blue = DAPI stained nuclei.

**Figure 6:**
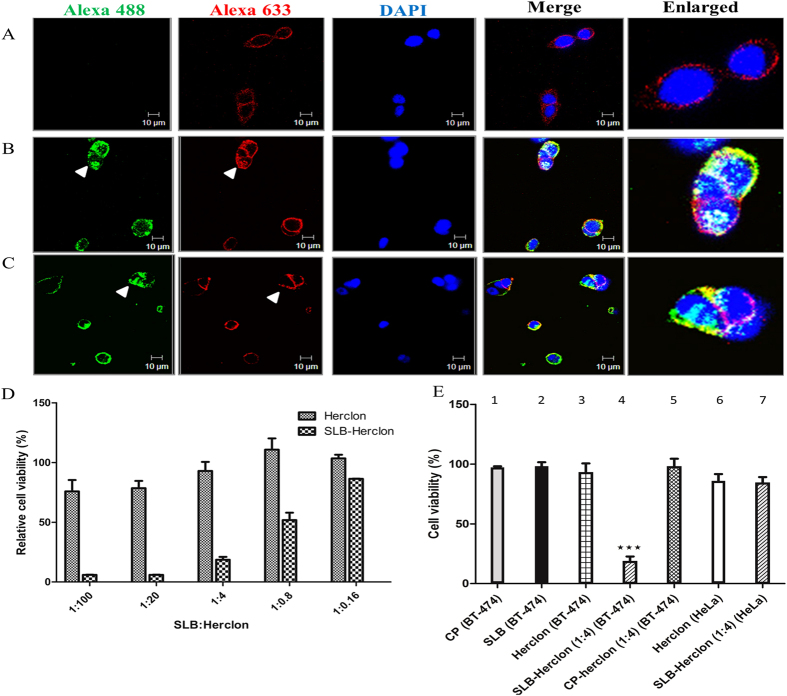
SLB mediated Herclon delivery in BT-474 cells. (**A**) Confocal images of BT-474 cells incubated with Herclon 633 (46 nM) for 1 hour. Confocal images of BT-474 cells treated with (**B**) SLB 488-Herclon 633 and (**C**) CP 488-Herclon 633 for 1 hour. White arrow represents enhanced cytoplasmic localization of SLB 488 and Herclon 633 in (**B**) and cytoplasmic localization of CP 488 and membrane localization of Herclon 633 in (**C**). Green = SLB 488/CP 488, red = Herclon 633, blue = DAPI stained nuclei, merge = combination of all three fluorophores and enlarged = enlarged version of merged image. (**D**) Cell viability assay of BT-474 cells incubated with varying concentration of Herclon (16.6 nM–0.026 nM) with and without SLB (0.166 nM) for 48 hours. (**E**) Cell viability assay of BT-474 cells incubated with CP (1.66 nM, bar 1) , SLB (1.66 nM, bar 2), Herclon (0.664 nM, bar 3), SLB-Herclon (1:4, bar 4) and CP-Herclon (1:4, bar 5) for 48 hours. Cell viability assay of HeLa cells when incubated with Herclon (0.664 nM, bar 6) and SLB-Herclon (1:4, bar 7), ***p < 0.05

The only antibody delivering vector (apart from gene therapy or chemical methods) reported thus far is the hemagglutinating virus of Japan envelope (HVJ-E)^10^, which is an animal viral vector that can deliver functional antibodies like anti-tubulin antibodies and anti- nuclear pore complex antibodies. SLB could deliver three different monoclonal antibodies targeted to plant toxin (abrin), intracellular tubulin or HER2 receptor intracellularly in various mammalian cells (Fig. 1D). D6F10 delivered by SLB was more effective in rescuing abrin mediated protein synthesis inhibition and apoptosis as compared to the current method of pre-incubation of D6F10 with abrin. Anti-tubulin antibodies have been delivered by lipopolyamines^23^, microinjection^40^ or via electroporation^41^. So far no plant VLPs have been shown to deliver anti-tubulin antibodies intracellularly. SLB is the first chimeric plant VLP shown to deliver anti-tubulin antibodies intracellularly and disrupt tubular network. The only other nanoparticle used for intracellular delivery of anti-tubulin antibodies is DOGS^23^.The aggregation pattern of tubulin upon interaction with the antibody was similar to that observed in this study. Herclon delivered by gold nanoparticles, showed only a 2 fold enhancement (~40%) in cytotoxicity^42^. Recently, it was shown that PVX conjugated with Herceptin results in a maximum of 22% cytotoxicity at a high concentration of Herceptin (20 μg)^15^. In contrast, SLB mediated Herclon delivery showed high toxicity (83%) which has not been observed with any other nanoparticle mediated antibody delivery.

There are various advantages of using SLB as a nanocarrier. Firstly, SLB has multiple B domains in its VLP and thus binds to IgGs with higher affinity than protein A. Secondly, antibody binding to B domain is a strong interaction and SLB could be easily bound with antibodies (incubation for 1 hr is sufficient) as compared to other known nanoparticles that employ tedious methods of loading antibodies^43^,^44^. Thirdly, no apparent toxicity was observed in cell viability assays with CP and SLB in HeLa and BT-474 cells *in vitro*. Further, the lack of antibody displacement in presence of sheep serum renders it a powerful tool for specific antibody delivery. Such an intracellular delivery of antibodies overcomes one of the major challenges in antibody therapy as most antibodies fail to cross cell membrane, widening the horizons of antibody based therapeutics. Similar studies with other therapeutic antibodies could lead to increase in the therapeutic efficiency of antibodies. Further research is in progress for targeted delivery of antibodies via SLB *in vivo*. Demonstration of SLB mediated delivery of different kinds of antibodies, targeting cellular as well as surface exposed antigens, substantiates the potential of SLB as a universal antibody nanocarrier in antibody based therapeutics.

## Methods

### Cloning, expression and purification of chimeric VLPs with B domain

For cloning the B domain in βH-βI loop region of SeMV CP, first the nucleotides corresponding to Ser 242 were mutated to alanine using CP sdm AfeI sense and antisense primers (Table 1) with pRSETC CP as template, such that a new restriction site AfeI was generated. The resultant plasmid (pRSETC CP sdm) was digested with AfeI enzyme. B domain gene, PCR amplified using pRSETC NΔ65B CP^45^ as template and B domain specific primers (Table 1), was ligated to AfeI digested pRSETC CP sdm to form pRSETC SLB His. For removal of the N terminal Histidine tag, the entire construct was PCR amplified using CP specific primers (Table 1) digested with EcoRI and inserted in NdeI end filled and EcoRI cut pRSETC vector. The clones (pRSETC SLB) were confirmed by PCR amplification using CP specific primers. All the three dimensional structures were rendered by Pymol^46^.

The recombinant proteins were over-expressed and purified from *E. coli* Rosetta cells transformed with pRSETC CP and pRSETC SLB. The transformed cells were grown in 500 ml LB broth at 37 °C till O.D._600_ reached 0.4, followed by addition of 0.3 mM IPTG and incubated at 16 °C for 12 hours. Cells were harvested and resuspended in ice cold 50 mM Tris HCl pH 7.5 containing 0.1% Triton X-100. After sonication, cell debris was removed by centrifugation of the suspension at 13,000 rpm, 30 min, 4 °C. The supernatant was ultrapelleted at 26,000 rpm, 3 hours, 4 °C using a SW32 rotor (Beckman Coulter). The ultrapellet was resuspended in 2 ml of 50 mM Tris HCl pH 7.5 by keeping on an end-to-end rotor overnight at 4 °C. The supernatant obtained after low speed centrifugation was layered on 10–40% sucrose density gradient and centrifuged at 26,000 rpm for 3 hours at 4 °C and analysed by SDS PAGE. The ultrapellet of the peak fractions were dissolved in 50 mM Tris HCl pH 7.5, 5% glycerol. Western blot analysis of purified CP and SLB were done using anti-CP (specific) and anti-DAPAL (non-specific) antibodies using protocol mentioned in^47^.

### Transmission electron microscopy

0.2 mg/ml protein was adsorbed on formvar coated copper grids (SPI Supplies, USA, Code:3440C-MB) for 2 min followed by wash in filter sterile 50 mM Tris-HCl buffer pH 7.5 for 30 sec. The grids were finally stained with filter sterilized 1% uranyl acetate for 1 min followed by buffer wash and then air dried for 4 hours. The grids were viewed in Tecnai G2 Spirit (Biotwin FEI, USA) electron microscope at 120 kx. The diameter of 50 particles was measured and analysed using ImageJ software.

### Direct antigen coating Enzyme Linked Immunosorbent Assay (DAC ELISA)

Varying concentration of VLPs (1-10000 nM (assuming 180 subunits)) were coated on nunc immune modules and incubated at 4 °C overnight. Similar concentration range of SpA (Protein A) was used as positive control and 100 μl phosphate buffered saline pH 7.5 (1 × PBS) was used as negative control. Following three times wash with 1 × PBS, all wells were blocked with 5% skimmed milk in 1 × PBS buffer for 2 hours at 37 °C. Henceforth, three times wash with 1 × PBST (1 × PBS containing 0.05% Tween-20) and 1 × PBS was followed after each step. Anti-DAPAL antibody (1:3000) was incubated for 1 hour. For detection, 1:7500 dilution of goat anti rabbit IgG HRP conjugate (Genei) was used. The reaction was stopped after significant colour development using 100 μl 2 N H_2_SO_4_. The absorbance was monitored in a microplate reader (Tecan infinite M200 pro) at 450 nm. The data obtained were plotted and analysed using GraphPad Prism software.

### Mammalian cells and their maintenance

The different kinds of mammalian cells used in this study include Human cervical cancer cells (HeLa), Keratinized HeLa (KB) (kind gift from Prof. Anjali Karande, IISc, India), *Mus musculus* melanoma (B16-F10) (kind gift from Prof. P. N. Rangarajan, IISc, India) and human breast carcinoma-derived cells (BT-474, ATCC No.HTB-20). Cancerous breast epithelial cells (CB) 704 and primary cultures of human mammary epithelial cells 704 (HMECs)^48^ were obtained from Prof Annapoorni Rangarajan, IISc, India. All cells were maintained in prescribed growth conditions. BT-474 cells were maintained in DMEM/F12 medium containing 1.2 g/L of sodium bicarbonate and 1 mM sodium pyruvate.

### Confocal microscopy

The proteins were labelled with Alexa 488/633 as per manufacturer’s protocol and purified labelled proteins were used for confocal microscopy as described earlier with minor modifications^19^. Briefly, 20,000 HeLa cells were adhered overnight on sterile coverslips and incubated with CP 488 (1.58 nM) for 2, 4, 8 and 10 hours in fresh DMEM media containing 10% FBS. The cells were washed twice with 1 × PBS pH 7.5, followed by fixation using 4% paraformaldehyde for 10 min. The cells were thoroughly washed, permeabilized with 1 × PBS buffer containing 0.01% saponin for 15 min and stained with DAPI (2.5 μg/μl) for 10 min. The cells were finally washed and mounted on coverslides in presence of Fluorosheild. Images were acquired using Plan-Neofluar 100x/1.3 oil objective of Zeiss 510 Meta microscope and analysed using Zeiss LSM image browser version 4.2.0.121. In order to check VLP entry in presence of non-specific proteins, CP 488 (1.58 nM) were added on HeLa cells for 2 hours along with BSA (10 μg) or sheep serum (40 ng/μl). To check the specificity of VLP entry, 10 nM of unlabelled CP was incubated along with 1.58 nM CP 488 for 2 hours. Chimeric SLB 488 was also incubated in HeLa cells in a similar manner. SLB 488 entry was also checked with KB, B16-F10, BT-474, CB 704 and HMECs 704 cells using the same protocol. The concentration of VLP and antibodies were fixed based on preliminary concentration variation experiments.

D6F10 internalization was monitored by initial pre incubation of SLB 488 (1.58 nM) with D6F10 633 (6.6 nM) for 1 hour followed by addition onto HeLa cells for 4 hours. D6F10 633 (6.6 nM) alone was used as negative control. Cells were washed, fixed, permeabilized and stained with DAPI as mentioned earlier. Time course analysis of SLB 488 -D6F10 633 entry was also performed in a similar manner for 2, 4, 8 and 12 hours. To check whether D6F10 could be displaced from the complex in presence of other antibodies, SLB 488-D6F10 633 was incubated in adhered HeLa cells for 3 hours along with sheep serum (40 ng/μl) and processed as mentioned earlier.

Similar protocol was followed for examining FITC labelled monoclonal anti-α-tubulin antibody delivery using SLB in HeLa cells. Fixed cells were permeabilized and probed with the antibody (30 ng/μl) to demonstrate the specificity of the anti-tubulin antibody. FITC labelled tubulin antibody (7.5 ng/μl) and (30 ng/μl) were pre-incubated with unlabelled SLB (1.58 nM) for 1 hour followed by addition on HeLa cells for 2 hours. SLB mediated delivery of tubulin antibody (7.5 ng/μl) was also monitored at varying time points (2, 4, 6 hours).

Herclon internalization was tested on adhered BT-474 cells using SLB 488 (1.58 nM) preincubated with Herclon 633 (46 nM) for 1 hour, followed by addition in adhered BT-474 cells for 1 hour in assay media (DMEM/F12 with 2 mM glutamine, 1.2 g/L of sodium bicarbonate, 3.15 g/L of glucose, 15 mM HEPES, 1 mM sodium pyruvate, and 2% FBS). Similar assay was also monitored for varying time points (1–3 hours) in presence of assay media. CP 488-Herclon 633 (1.58 nM, 46 nM) and Herclon 633 alone (46 nM) were used as controls. BT-474 cells after each treatment were processed in a similar manner as mentioned above. Similar assay was performed in adhered HeLa cells using Herclon 633 (46 nM) as well as SLB 488-Herclon 633 (1.58 nM, 46 nM) for 2 hours.

### Protein synthesis assay using Tritiated Leucine

Translation assays were performed as reported in S. Bagaria *et al.*^19^ using abrin (0.166 nM) and abrin pre-incubated with varying concentration of D6F10 (abrin: D6F10 = 1:25, 1:50) as controls. SLB (1.5 nM) pre incubated (1 hour) with D6F10 were incubated with HeLa cells for 2 hours. After washing with PBS, abrin treatment was done for 7 hours in all assays. The DMEM media was replaced with RPMI Leucine free medium for 2 hours and pulse chased with [^3^H] leucine (0.4 μCi) for 2 hours. Overnight precipitation was carried in presence of 5% TCA at 4 °C. The precipitate was washed once with 200 μl of 20% ethanol, dried and dissolved in 1% SDS, 0.1 N NaOH and incubated with 4 ml scintillation liquid (Cocktail T, Spectrobiochem) for 5 hours. Radioactivity was measured using Beckman scintillation counter. To ensure that internalization of SLB or CP did not affect protein synthesis, the respective proteins were used as control. Buffer treated cells are referred to as untreated sample. CP (1.5 nM) pre-incubated (1 hour) with D6F10 (75 nM) was also used as a negative control.

### Cell cycle progression analysis

For cell progression analysis, 60,000 cells were incubated with CP (10 nM) and SLB (10 nM) for 36 hours. In order to check whether SLB-D6F10 can rescue the effect of abrin induced apoptosis, HeLa cells were treated with abrin (0.16 nM) and abrin-D6F10 (molar ratio = 1:50) for 36 hours. SLB (0.1 nM)-D6F10 (5 nM) (pre-incubated for 1 hour) were incubated for 4 hours, washed with PBS twice, followed by abrin treatment for 36 hours. All cells were trypsinized, washed and then fixed using 1 ml 70% ethanol at −20 °C overnight. The cells were pelleted, washed thrice with ice cold PBS and treated with RNase (10 μg/ml, Sigma) for 30 min followed by 15 min incubation with Propidium Iodide (0.3 μg/μl, Sigma). For each reaction, 10,000 events for single cells were recorded in triplicates. All data were acquired by BD FACS Verse (Propidium iodide: Excitation −488 nm, Emission-586 ± 21 nm) and analysed by BD FACS Diva software.

### Cell viability assay

The biological activity of Herclon was tested using anti-proliferation assay by resazurin sodium staining method^49^. Briefly, 0.1 million BT-474 cells/ml were adhered in 96 well plates (Nunc immunomodules) in assay media (DMEM/F12 with 2 mM glutamine, 1.2 g/L of sodium bicarbonate, 3.15 g/L of glucose, 15 mM HEPES, 1 mM sodium pyruvate, and 2% FBS). BT-474 cells were treated with varying concentrations of Herclon 100 μl (16.6 nM–0.026 nM) (diluted in assay medium) alone or preincubated for 1 hour with SLB (0.166 nM), in a humidified incubator at 37 °C with 5% CO_2_ for 48 hours, followed by addition of 30 μL of pre-warmed resazurin sodium (Invitrogen) (10×) to each well. After 7 hours, the plate was cooled to room temperature and fluorescence was measured at an excitation wavelength of 530 nm and emission wavelength of 590 nm. The percentage of cell viability was calculated as the fraction of fluorescence (sample)/ fluorescence (untreated cells), multiplied by 100. Similar assay was performed in BT-474 cells with CP (1.66 nM), SLB (1.66 nM), Herclon (0.664 nM), SLB-Herclon (1:4) and CP-Herclon (1:4) at 48 hours. As controls, Herclon (0.664 nM) and SLB-Herclon (1:4) was added in adhered HeLa cells and incubated for 48 hours and cell viability was analysed by addition of resazurin sodium as mentioned earlier.

### Statistical analysis

For protein synthesis assay, cell cycle progression assay and anti-proliferation assay, ANOVA followed by Tukey’s multiple comparison tests was performed in GraphPad Prism version 5.00 for Windows, GraphPad Software, San Diego California USA, ( www.graphpad.com). P values less than 0.05 were considered to be statistically significant. All values are expressed as mean ± standard deviation (S.D.).

## Additional Information

**How to cite this article**: Abraham, A. *et al.* Intracellular delivery of antibodies by chimeric Sesbania mosaic virus (SeMV) virus like particles. *Sci. Rep.* **6**, 21803; doi: 10.1038/srep21803 (2016).

## Supplementary Material

Supplementary Information

## Figures and Tables

**Table 1:** Primers used in this study.

Primer name	Sequence	Length	Description
CP sdm AfeI sen	GCTCTGCTGGATGGGTCGAGCGCTACAGCTGTGGCTGCTGGAC	43	Used for creating AfeI site on SeMV CP βH-βI loop via site directed mutagenesis
CP sdm AfeI anti	CGAGACGACCTACCCAGCTCGCGATGTCGACACCGACGACCTG	43	
B sen	GATAACAAATTTAACAAAGAACAGC	25	PCR amplification of B domain to be cloned at the βH-βI loop
B anti	TTCTTTCGGCGCCTGCGCAT	20	
CP sen	ATGGCGAAAAGGCTTTCGAAACAACAG	27	PCR amplification of SLB from pRSETC SLB His for creating pRSETC SLB
CP anti	CAAGAATTCGGTACCTCAGTTGTTCAGGGC	30	

Sen and anti refers to the sense and antisense primers. The underlined nucleotides in CP sdm AfeI primers refers to the altered nucleotides that replace TTCC in pRSETC CP.
