# Supplementary material for: Intracellular delivery of antibodies by chimeric Sesbania mosaic virus (SeMV) virus like particles
Source: Sci Rep. 2016 Feb 24;6:21803. doi: 10.1038/srep21803 (PMC4764859; doi:10.1038/srep21803)
Supplement: Supplementary Information [file srep21803-s1.doc]

**Intracellular delivery of antibodies by chimeric Sesbania mosaic virus (SeMV) virus like particles**

Ambily Abraham^1^, Usha Natraj^1^, Anjali A. Karande^1^, Ashutosh Gulati^2^, Mathur R. N. Murthy^2^, Sathyabalan Murugesan^3^, Pavithra Mukunda^3^ and Handanahal S. Savithri^1,*^

^1^Department of Biochemistry, Indian Institute of Science, Karnataka, India

^2^Molecular Biophysics Unit, Indian Institute of Science, Karnataka, India

^3^Theramyt Novobiologics Pvt. Ltd., Karnataka, India

^*^[bchss@biochem.iisc.ernet.in](mailto:bchss@biochem.iisc.ernet.in)


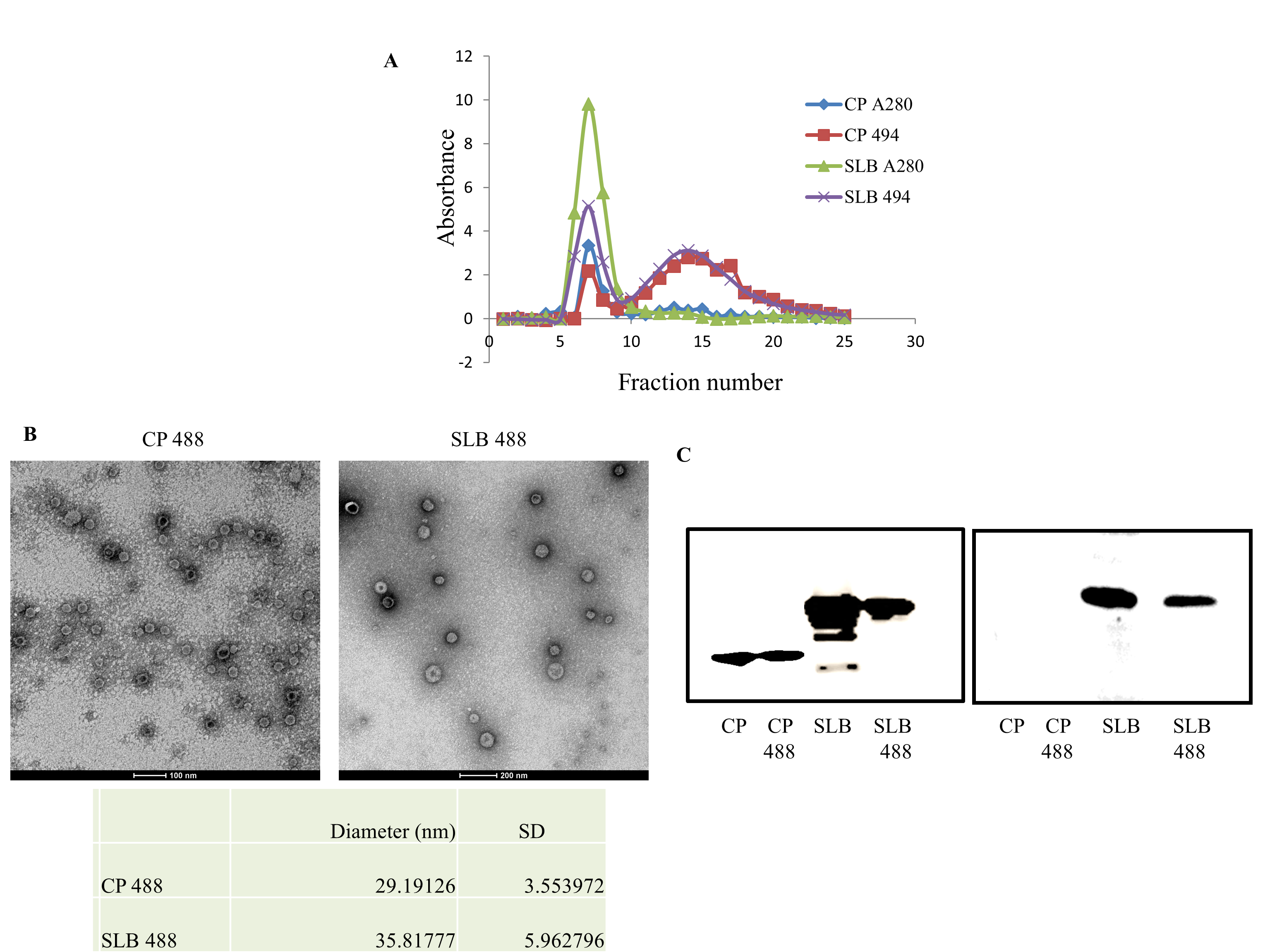


**Supplementary Figure S1: Biochemical analysis of Alexa Fluor 488 labeled VLPs.** (A) Elution profile of CP 488 and SLB 488 after labeling with Alexa Fluor 488. Absorbance at 280 and 494 of the respective proteins are represented. (B) Electron micrographs of CP 488 and SLB 488. The table represents the average diameter of 50 capsids and its standard deviation analyzed using ImageJ software. (C) Western blot analysis of CP, CP 488, SLB and SLB 488 using CP polyclonal antibody (left blot) and DAPAL polyclonal antibody (right blot).


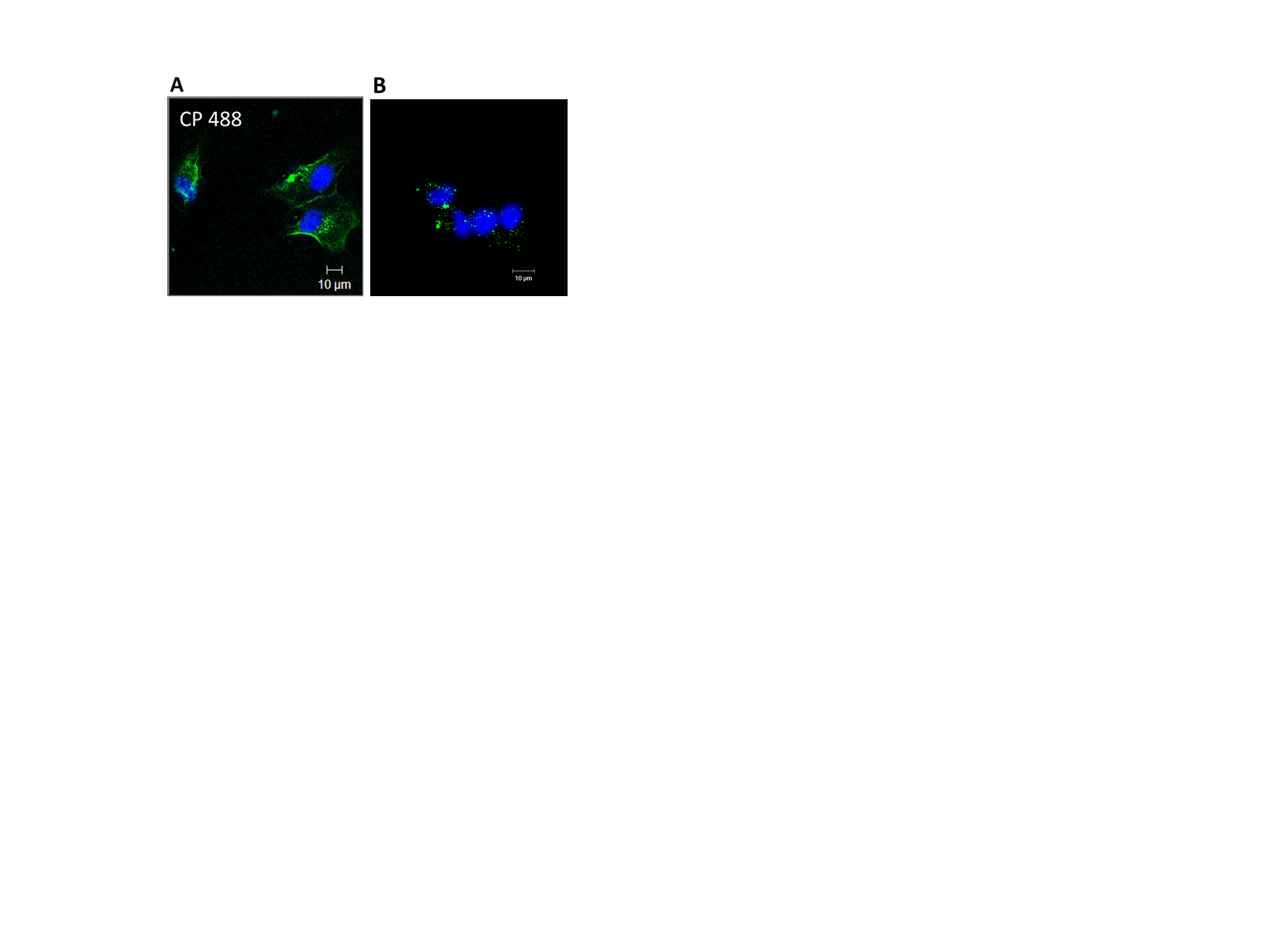


**Supplementary Figure S2: Specificity of CP 488 entry in HeLa cells.** Confocal images of HeLa cells treated with 1.58 nM CP 488 for 2 hours at 37 °C in the absence (A) and in the presence of 10 nM CP (unlabeled) (B). Green=CP 488, Blue=DAPI stained nucleus


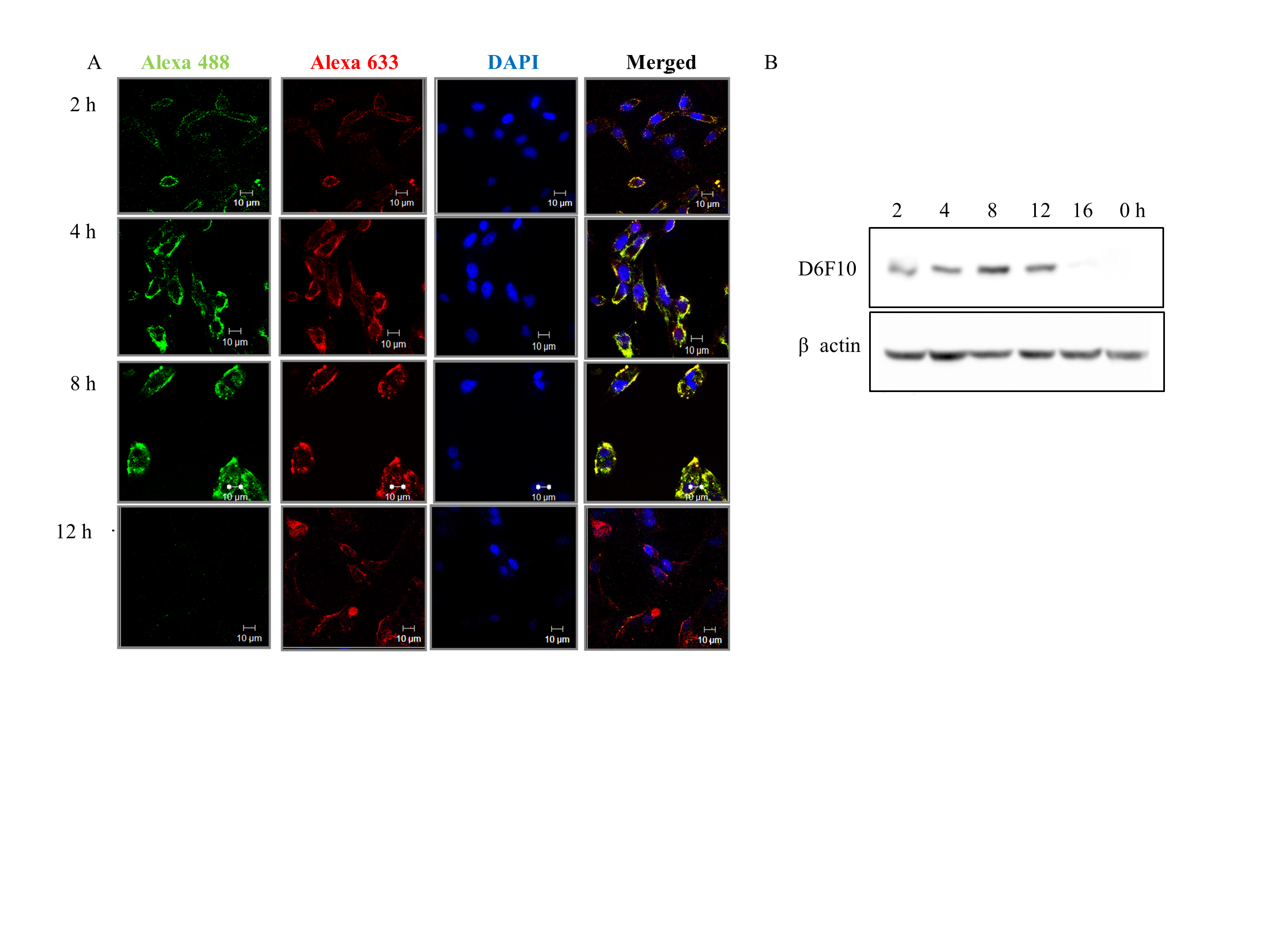


**Supplementary Figure S3: Kinetics of SLB mediated D6F10 delivery in HeLa cells.** SLB 488 pre-incubated with D6F10 633 was incubated with adhered HeLa cells for 2, 4, 8 and 12 hours and processed for confocal microscopy as mentioned in methods section. Green = SLB 488 (first column), red = D6F10 633 (second column), blue = DAPI stained nuclei (third column) and merge of first three columns is shown in the last column.


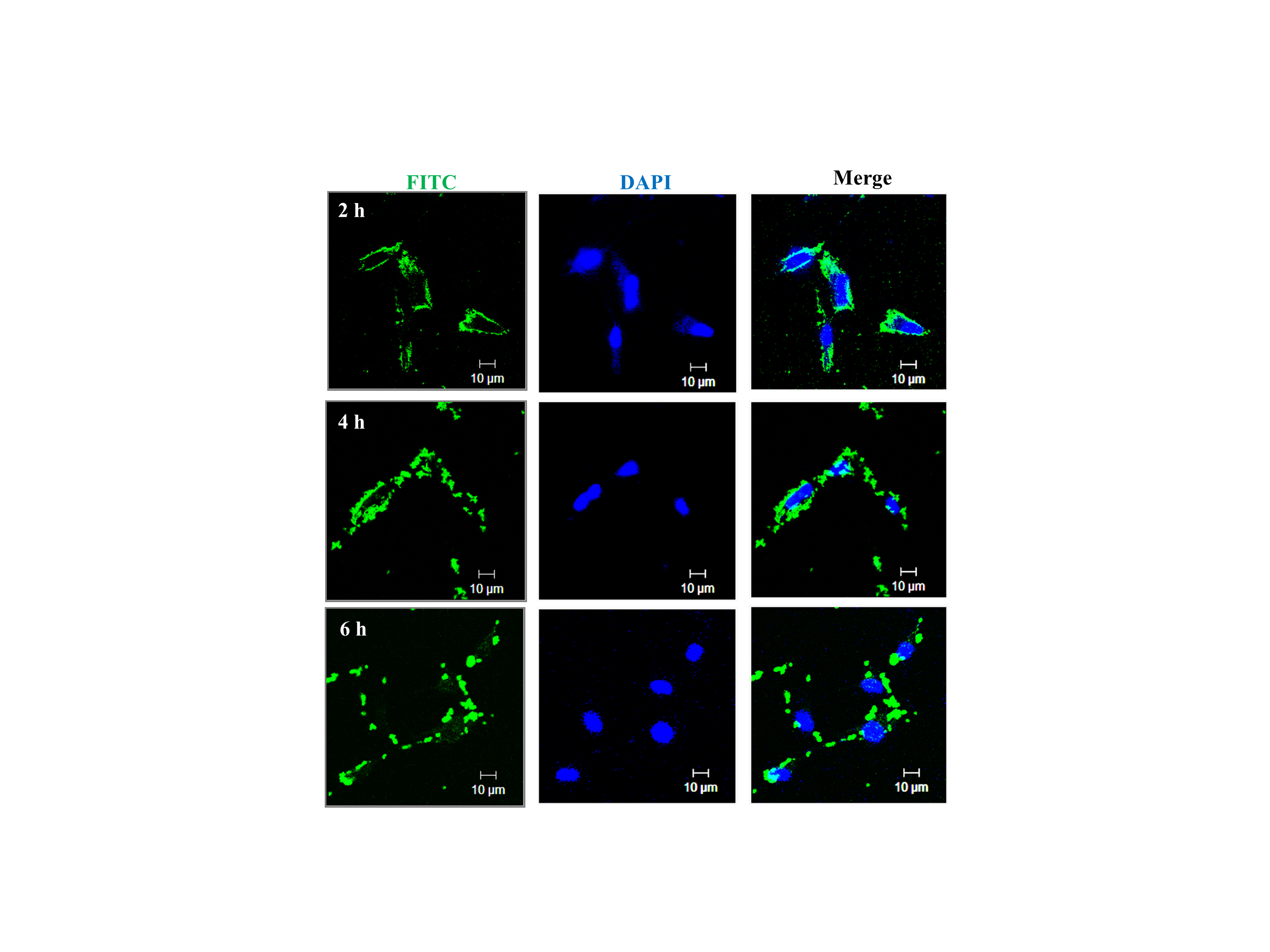


**Supplementary Figure S4: Kinetics of SLB mediated anti-tubulin antibody delivery.** Confocal images of anti-α-tubulin antibody (1:200) preincubated with SLB and later incubated with HeLa cells for 2 hours (top row), 4 hours (middle row) and 6 hours (last row). Green=FITC labeled anti- tubulin antibody, blue=DAPI stained nuclei, Merge=overlay of the first 2 images.


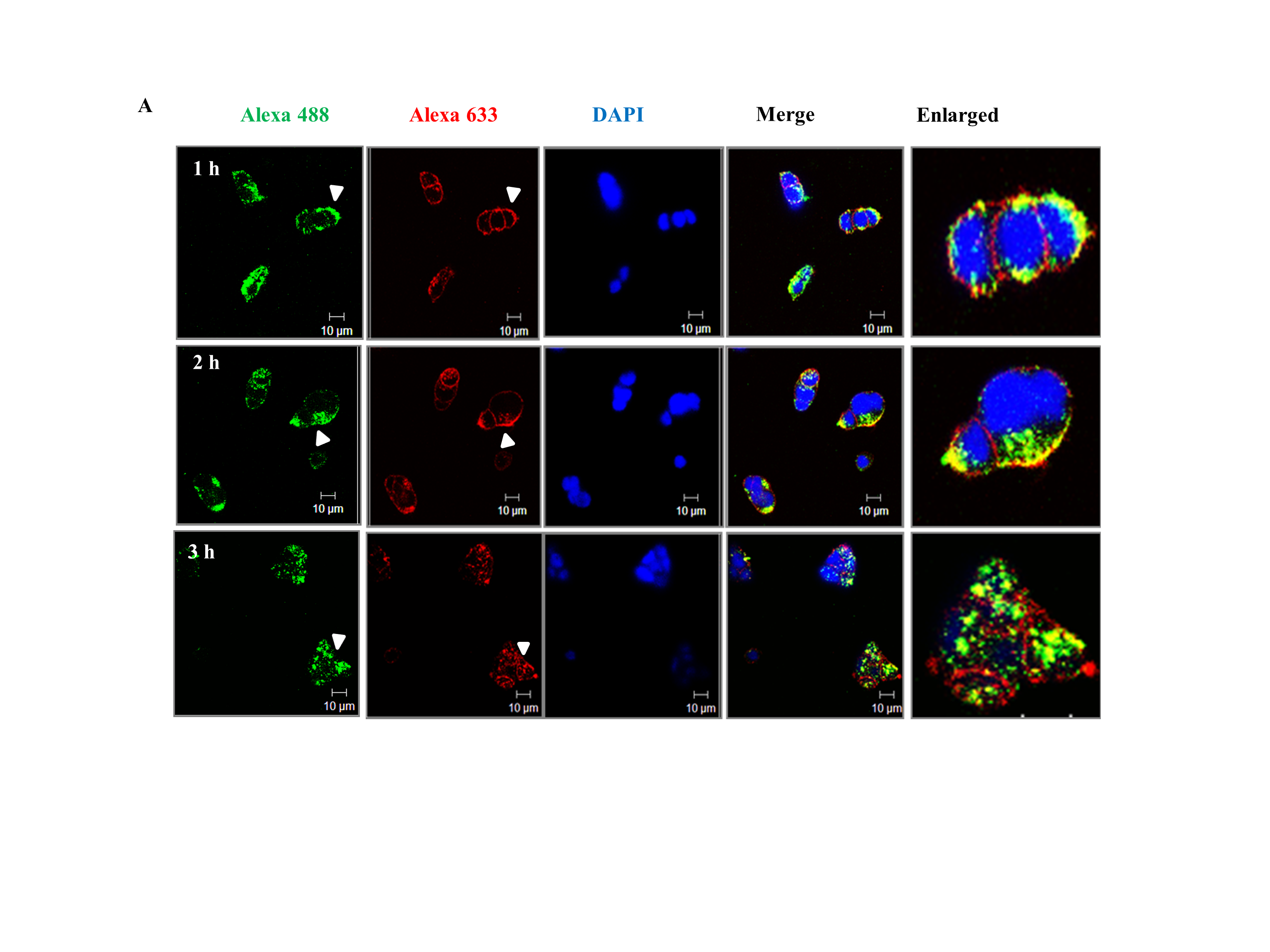


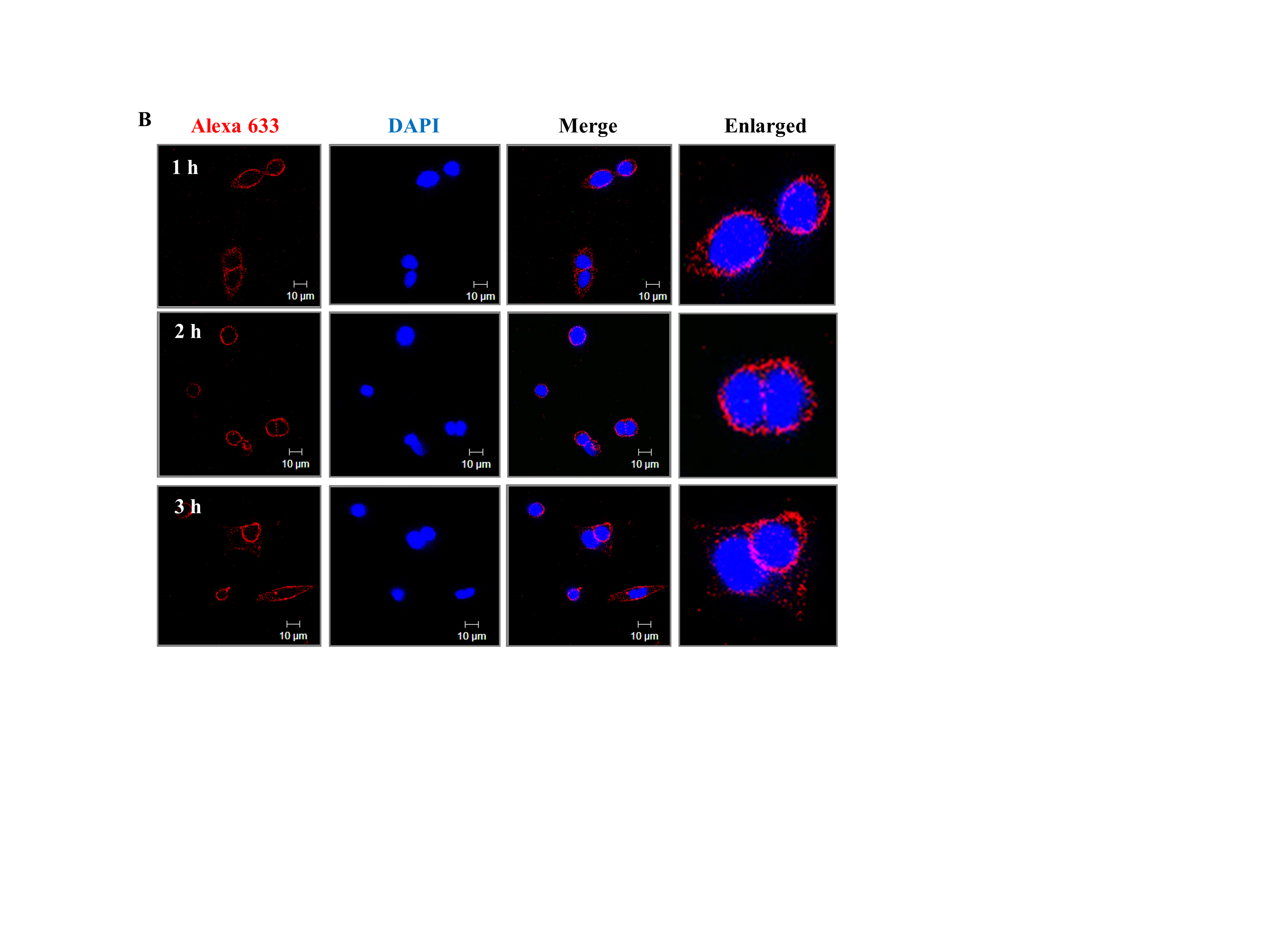


**Supplementary Figure S5: Kinetics of SLB mediated Herclon delivery in BT-474 cells.** A) Confocal microscopic images of time course of 46 nM Herclon 633 entry when preincubated with 1.58 nM SLB for 1 hour (top row), 2 hours (middle row) and 3 hours (last row) as mentioned in the methods section. The different columns represent the fluorescence label used (mentioned at the top of the column). B) Similar assay using Herclon 633 (46 nM) alone.


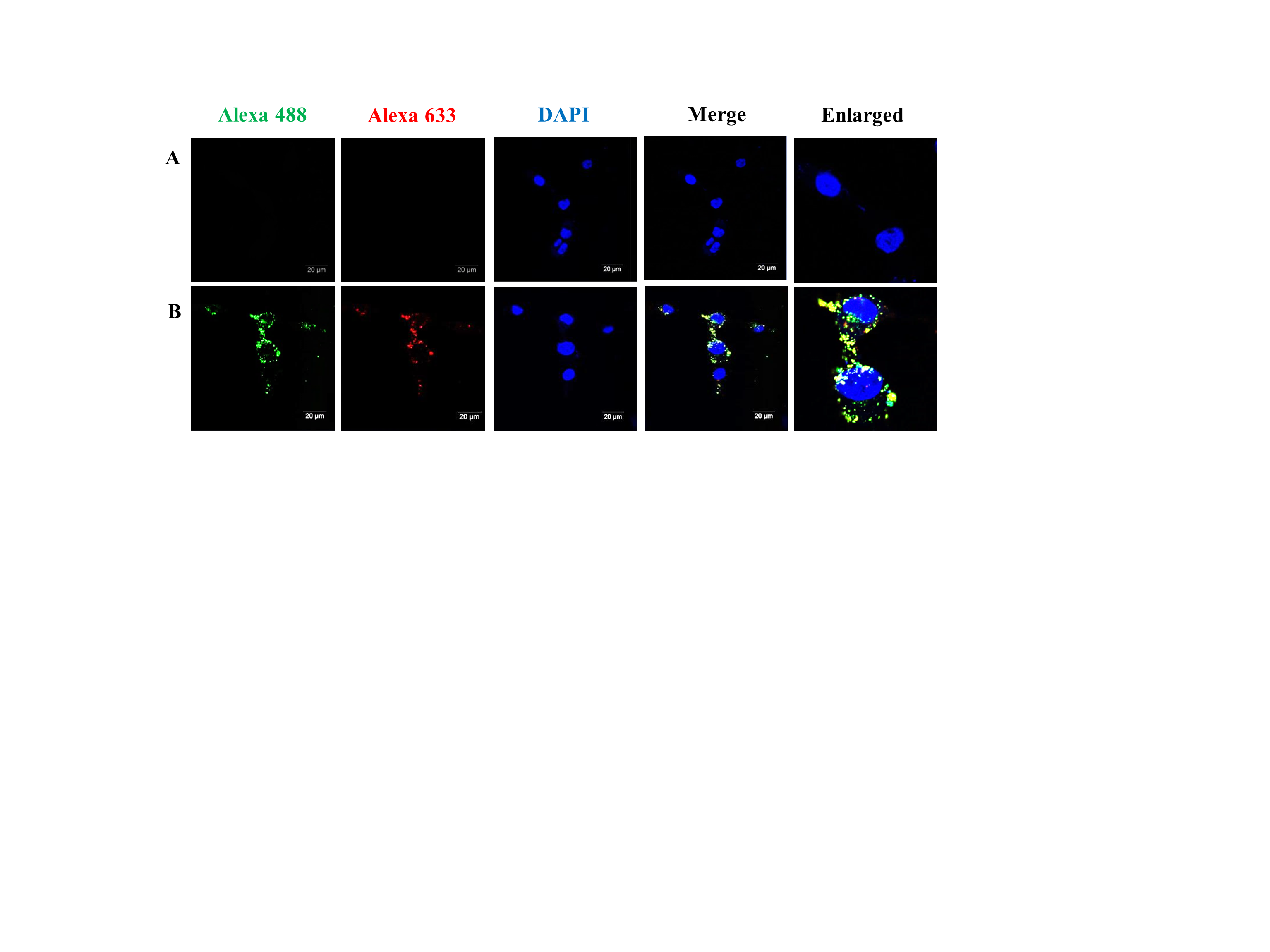


**Supplementary Figure S6: SLB mediated Herclon delivery in HeLa cells.** Confocal microscopic images acquired using Zeiss LSM 880 with Airyscan of HeLa cells incubated with A) 46 nM Herclon 633 and B) SLB 488-Herclon 633 (1.58 nM, 46 nM) for 2 hours followed by cell fixation and DAPI staining. Green=SLB 488/CP 488, red= Herclon 633, blue= DAPI stained nuclei, merge = combination of all three fluorophores and enlarged = enlarged version of merged image.
